# The Short-Chain Fatty Acids Propionate and Butyrate Augment Adherent-Invasive Escherichia coli Virulence but Repress Inflammation in a Human Intestinal Enteroid Model of Infection

**DOI:** 10.1128/Spectrum.01369-21

**Published:** 2021-10-06

**Authors:** Fernanda Pace, Sara E. Rudolph, Ying Chen, Bin Bao, David L. Kaplan, Paula I. Watnick

**Affiliations:** a Division of Infectious Diseases, Boston Children’s Hospital, Boston, Massachusetts, USA; b Department of Pediatrics, Harvard Medical School, Boston, Massachusetts, USA; c Department of Biomedical Engineering, Tufts University, Medford, Massachusetts, USA; d Division of Gastroenterology, Hepatology and Nutrition, Boston Children’s Hospital, Boston, Massachusetts, USA; Wayne State University

**Keywords:** inflammation, short-chain fatty acid, adherent-invasive *Escherichia coli*, butyrate, cell invasion, propionate

## Abstract

Short-chain fatty acids (SCFAs), which consist of six or fewer carbons, are fermentation products of the bacterial community that inhabits the intestine. Due to an immunosuppressive effect on intestinal tissue, they have been touted as a therapeutic for inflammatory conditions of the bowel. Here, we study the impact of acetate, propionate, and butyrate, the three most abundant SCFAs in the intestine, on gene expression in the intestinal pathobiont adherent-invasive Escherichia coli. We pair this with adherence, invasion, and inflammation in Caco-2 and human intestinal enteroid (HIE)-derived monolayer models of the intestinal epithelium. We report that propionate and butyrate upregulate transcription of adherent-invasive Escherichia coli (AIEC) flagellar synthesis genes and decrease expression of capsule assembly and transport genes. These changes are predicted to augment AIEC invasiveness. In fact, SCFA supplementation increases AIEC adherence to and invasion of the Caco-2 monolayer but has no effect on these parameters in the HIE model. We attribute this to the anti-inflammatory effect of propionate and butyrate on HIEs but not on Caco-2 cells. We conclude that the potential of SCFAs to increase the virulence of intestinal pathogens should be considered in their use as anti-inflammatory agents.

**IMPORTANCE** The human terminal ileum and colon are colonized by a community of microbes known as the microbiota. Short-chain fatty acids (SCFAs) excreted by bacterial members of the microbiota define the intestinal environment. These constitute an important line of communication within the microbiota and between the microbiota and the host epithelium. In inflammatory conditions of the bowel, SCFAs are often low and there is a preponderance of a conditionally virulent bacterium termed adherent-invasive Escherichia coli (AIEC). A connection between SCFA abundance and AIEC has been suggested. Here, we study AIEC in monoculture and in coculture with human intestinal enteroid-derived monolayers and show that the SCFAs propionate and butyrate increase expression of AIEC virulence genes while concurrently bolstering the intestinal epithelial barrier and reducing intestinal inflammation. While these SCFAs have been promoted as a therapy for inflammatory bowel conditions, our findings demonstrate that their effect on bacterial virulence must be considered.

## INTRODUCTION

The intestines of animals are in constant communication with the external environment and thus become densely populated with a diverse bacterial community. This community secretes a variety of short-chain fatty acids (SCFAs) through the fermentation of dietary polysaccharides left behind by the host (1). SCFAs are defined as fatty acids with fewer than 6 carbons and include acetate (C2), propionate (C3), and butyrate (C4), which are the most abundant SCFAs in the human intestine (2). The host response to these SCFAs promotes intestinal health and metabolic homeostasis. In particular, there is ample evidence that SCFAs provide energy to intestinal cells, promote the integrity of the epithelial barrier, and decrease inflammation (3, 4).

Intestinal concentrations of SCFAs depend on the composition of the intestinal microbial community, and this, in turn, depends on host genetic factors, host diet, and the extent of intestinal inflammation (5). Inflammation, in turn, can reshape the intestinal microbial community, leading to a preponderance of proteobacteria and, hence, altered levels of microbial metabolites (6–8). Although there are several drugs available for treatment of intestinal inflammation, one third of the patients either do not respond or develop unacceptable side effects (9). Therefore, there is a clinical need for new therapies. Because of their anti-inflammatory effects, SCFAs have been proposed as treatments for pathological conditions of the bowel that involve inflammation, including infection, malignancy, and idiopathic inflammatory bowel diseases such as Crohn’s disease and ulcerative colitis (10).

The proteobacterium Escherichia coli is a common inhabitant of the mammalian intestine. Although most E. coli strains have a commensal relationship with the host intestine, the genomes of adherent-invasive E. coli (AIEC) encode additional virulence factors that enable them to adhere to and invade intestinal cells (11). AIEC is more abundant in affected intestinal regions of some patients with Crohn’s disease and colorectal cancer (12–15). These patients also demonstrate a decrease in intestinal SCFAs (3). However, it is not clear whether AIEC plays a role in pathology, is favored by the pathological environment, or both (16).

Here, we have analyzed the impact of the SCFAs propionate and butyrate on AIEC gene expression, studied the effect of these SCFAs on AIEC adhesion and invasion of human adenocarcinoma-derived cell line Caco-2 and human intestinal enteroid (HIE)-derived monolayers, and then determined how these SCFAs affect transcription and expression of a panel of inflammatory and anti-inflammatory genes in these epithelial monolayer models. We find that, while propionate and butyrate promote AIEC virulence, they also induce an anti-inflammatory state in the HIE model that prevents increased AIEC adhesion and invasion. While our results support propionate and butyrate as therapies for inflammatory bowel disease, they also suggest that SCFAs may create an intestinal environment more conducive to epithelial invasion by bacterial pathogens.

## RESULTS

### Propionate and butyrate but not acetate increase AIEC adhesion and invasion of Caco-2 cells.

**FIG 1:**
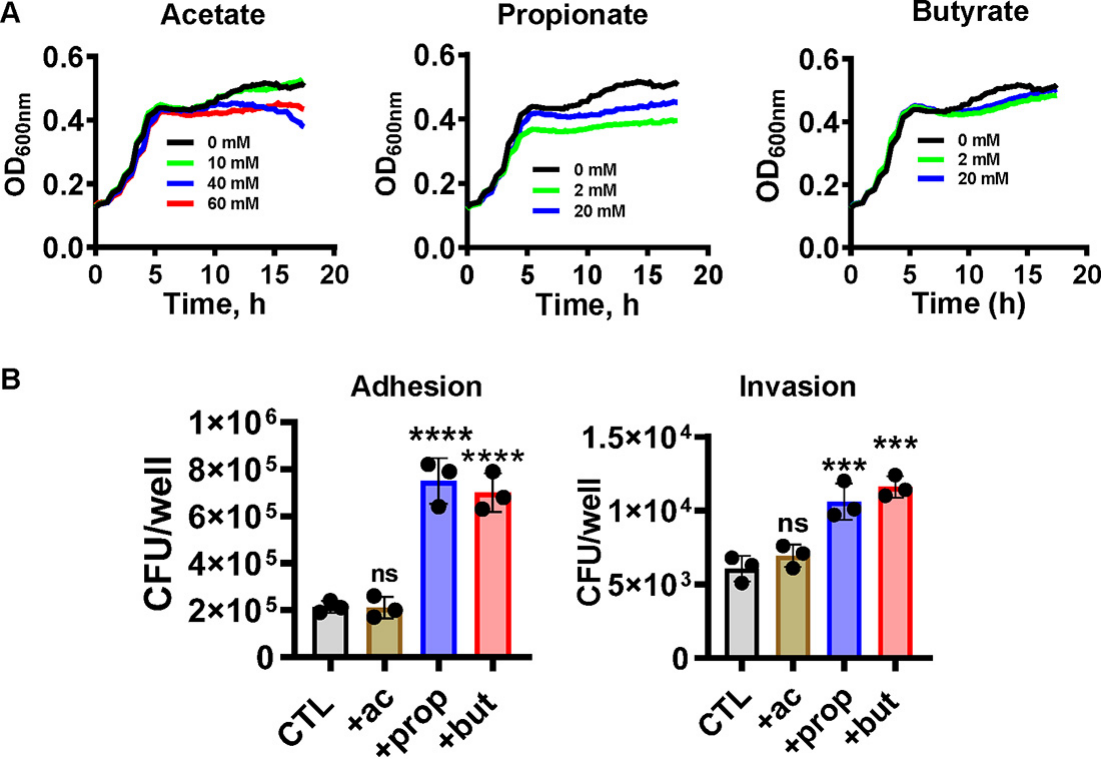
Supplementation with propionate or butyrate but not acetate increases AIEC adhesion to and invasion of Caco2 cells. (A) Growth of AIEC in DMEM, low glucose supplemented with the indicated concentrations of acetate, propionate, or butyrate. (B) AIEC adhesion to and invasion of Caco2 cells in the presence of 40 mM acetate, 20 mM propionate, or 20 mM butyrate. The mean of experimental triplicates is shown. Error bars represent the standard deviation. Significance was assessed by a one-way ANOVA with Dunnett’s multiple-comparison test. ****, *P* < 0.0001; ***, *P* < 0.001; ns, not significant.

We questioned whether SCFAs might increase the virulence characteristics of AIEC. As a control, we first examined growth of AIEC in Dulbecco’s modified Eagle medium (DMEM), low glucose alone or supplemented with various amounts of acetate, propionate, or butyrate. Acetate and propionate mildly inhibited the growth of AIEC, principally in stationary phase (Fig. 1A). We then measured the impact of 40 mM acetate, 20 mM propionate, or 20 mM butyrate on AIEC adhesion and invasion of the human colorectal adenocarcinoma cell line Caco-2 after 3 and 4 h of infection, respectively (Fig. 1B). These concentrations of acetate, propionate, and butyrate are similar to those found in the human cecum (2). Interestingly, propionate and butyrate but not acetate increased adhesion and invasion of AIEC, suggesting that these treatments might augment expression of virulence factors. Furthermore, because acetate did not have the effect of propionate or butyrate, we conclude that our observations cannot be attributed to the increased osmolarity of the SCFA-supplemented medium.

### Butyrate supplementation of AIEC modulates virulence-related gene expression in the absence of exposure to intestinal cells.

**FIG 2:**
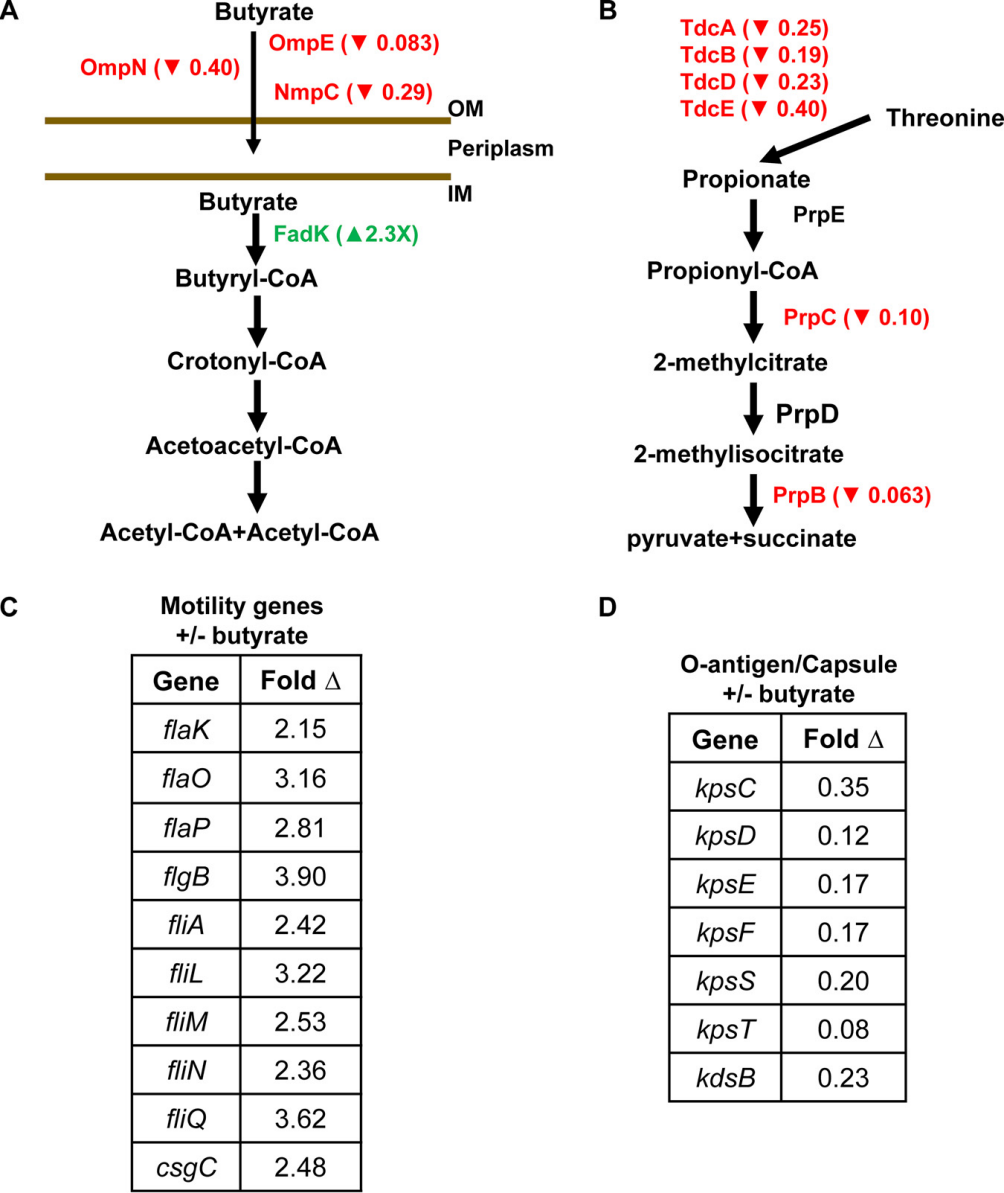
Butyrate supplementation alters the AIEC transcriptome. AIEC genes in the catabolic pathway of (A) butyrate and (B) propionate that are differentially regulated in response to supplementation with 20 mM butyrate. Arrows indicate an increase (▴) or decrease (▾) in transcription. (C and D) AIEC genes involved in (C) motility and (D) O-antigen and capsule synthesis whose transcription is differentially regulated by butyrate. The mean of experimental triplicates is shown. Fold change relative to the unsupplemented condition is reported. Significant measurements were required to have a false-discovery rate (FDR) of <0.1 and fold change of >2 or <0.5.

To assess the impact of butyrate on the AIEC transcriptome, we undertook an RNA-seq experiment under conditions comparable to those of our adhesion and invasion assays in the presence and absence of 20 mM butyrate (Table S1). Only 37 AIEC genes were significantly differentially regulated in response to butyrate supplementation. Genes encoding proteins involved in butyrate uptake were downregulated, while those involved in butyrate catabolism were upregulated, suggesting a direct response to butyrate supplementation (Fig. 2A). In addition, transcription of genes involved in threonine degradation to propionate and propionate catabolism was decreased (Fig. 2B). Two categories of differentially regulated genes that have previously been implicated in E. coli adhesion and invasion of mammalian cells were also identified. These include flagellar and pilus assembly genes, whose transcription was increased in the presence of butyrate (Fig. 2C), and group 2 capsule transport and assembly genes, whose transcription was decreased (Fig. 2D) (17–19).

To demonstrate a phenotypic correlate for the increased transcription of flagellar genes, we also assessed the impact of butyrate supplementation on motility. As shown in Fig. S1A, AIEC motility increased with butyrate supplementation. Although motility is also associated with increased biofilm formation, very little biofilm formation was observed under these conditions (Fig. S1B).

### Propionate and butyrate differentially regulate AIEC flagellar and capsule gene transcription while increasing expression of Tlr5 and TNF-α in infected Caco-2 monolayers.

**FIG 3:**
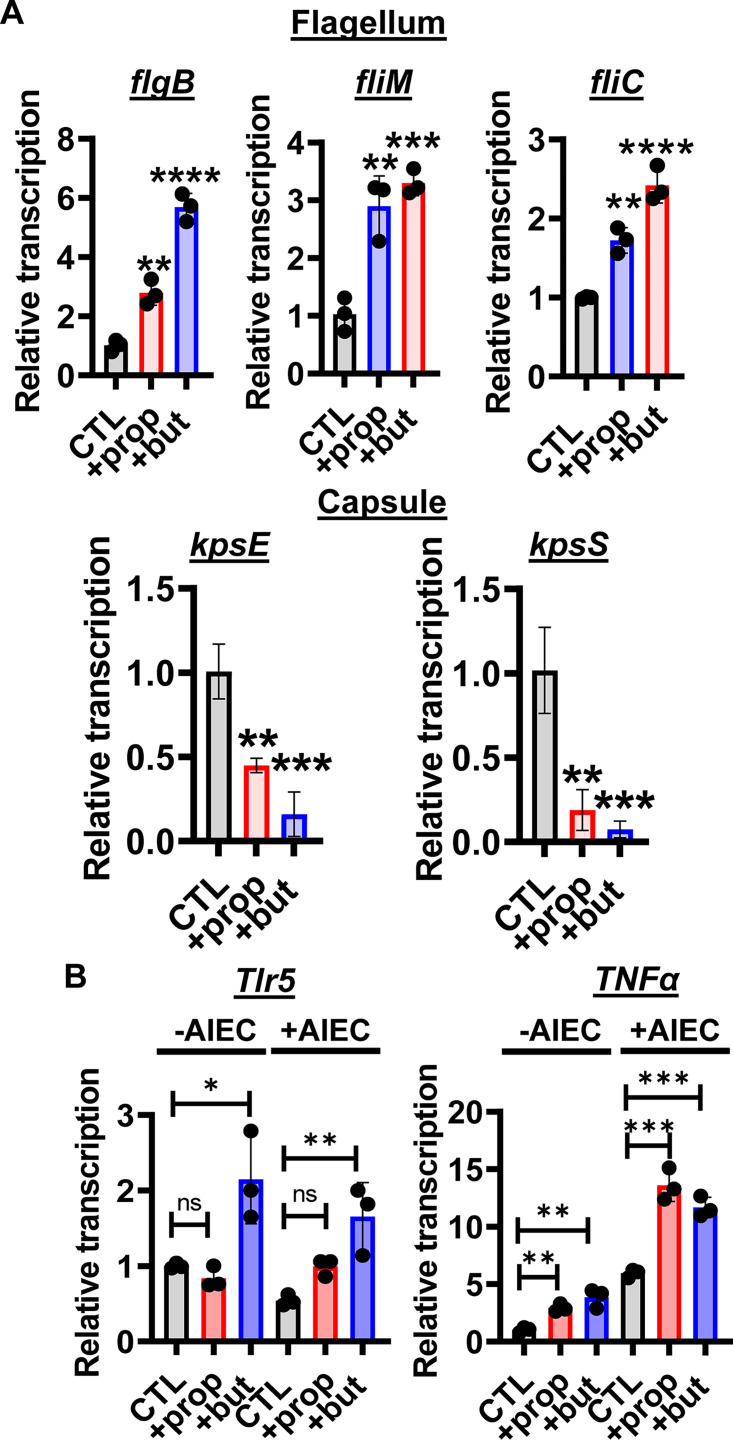
Propionate or butyrate supplementation of AIEC incubated with Caco-2 cells activates transcription of AIEC flagellar genes, and this correlates with increased transcription of *Tlr5* and *TNFa* in Caco-2 cells. (A) qRT-PCR transcriptional analysis of the indicated flagellar and capsule genes in AIEC cocultured with Caco-2 cells in DMEM, low glucose alone (CTL) or supplemented with 20 mM propionate (prop) or butyrate (but). (B) qRT-PCR analysis of *Tlr5* and *TNFa* transcription in Caco-2 cells alone (CTL) or treated with 20 mM propionate or butyrate in the presence or absence of AIEC as indicated. The mean of biological triplicates is indicated. Error bars represent the standard deviation. Significance was assessed by a one-way ANOVA with Dunnett’s multiple-comparison test. ****, *P* ≤ 0.0001; ***, *P* ≤ 0.001; **, *P* ≤ 0.01; *, *P* ≤ 0.05; ns, not significant.

We then used real-time quantitative reverse transcription PCR (qRT-PCR) to measure AIEC gene expression in the presence of Caco-2 monolayers with 20 mM propionate or butyrate supplementation. As shown in Figure 3A, both propionate and butyrate altered transcription of AIEC flagellar and capsular genes in the presence of Caco-2 monolayers, although the effect of propionate was smaller than that of butyrate. We did not detect an increase in transcription of curli and type I pilus synthesis genes (Fig. S1C).

Bacterial flagellin is recognized by the innate immune receptor Toll-like receptor 5 (Tlr5). We hypothesized that the increase in expression of AIEC flagellar genes might induce a coordinate increase in Caco-2 Tlr5 expression leading to greater signaling through NF-κB to activate tumor necrosis factor alpha (TNF-α) expression. In fact, we observed an increase in Tlr5 expression in response to butyrate but not propionate supplementation in both the presence and the absence of AIEC, suggesting that sensitization of Caco-2 cells to bacterial flagellin by this SCFA does not depend on flagellin. This was not observed for Tlr4, suggesting some Tlr specificity (Fig. S1D). Propionate and butyrate also increased expression of TNF-α both in the presence and absence of AIEC (Fig. 3B), demonstrating a proinflammatory effect of these SCFAs in the Caco-2 model.

### In an HIE-derived monolayer, propionate and butyrate oppose inflammatory cascades and promote epithelial integrity.

**FIG 4:**
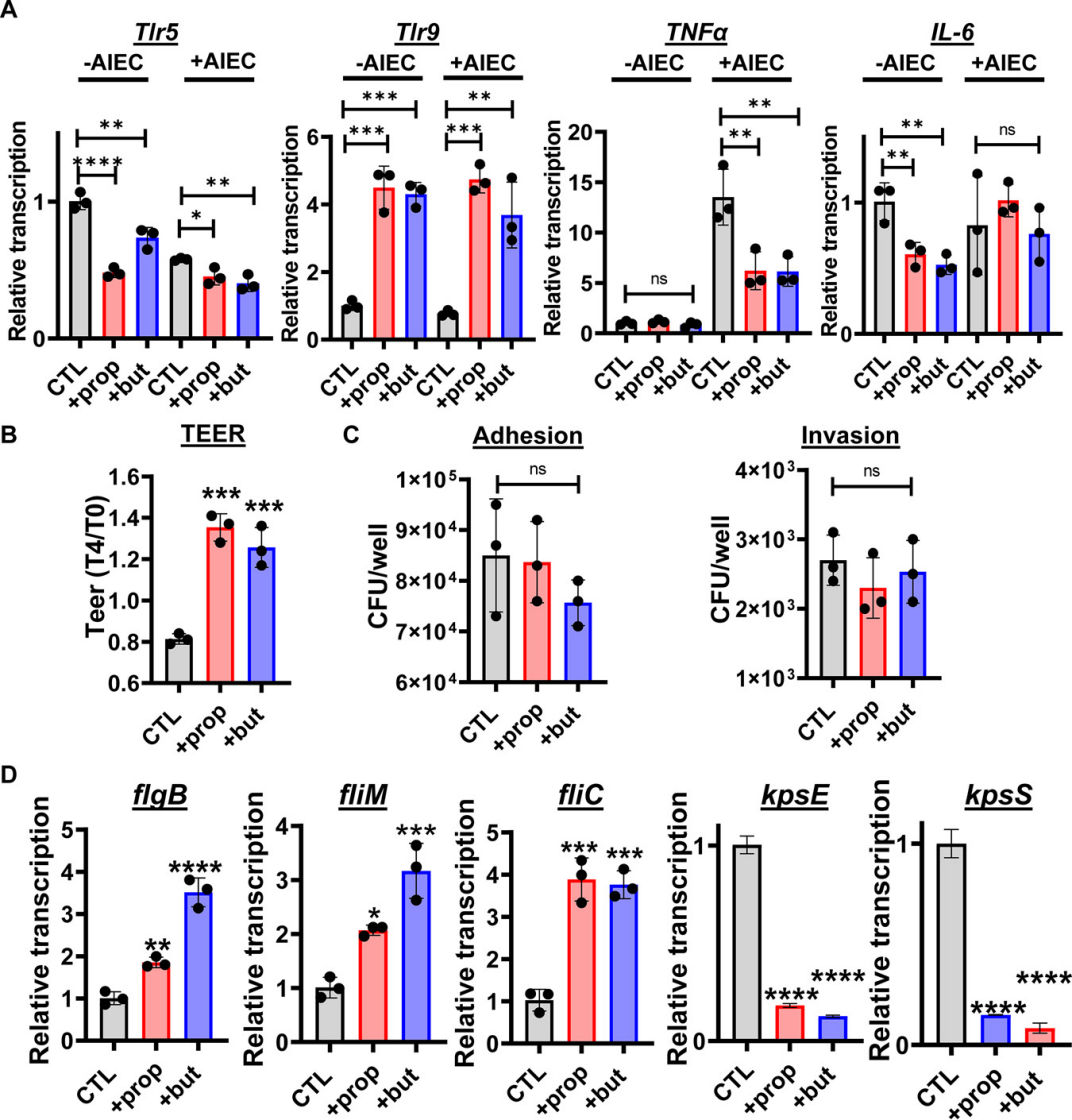
Propionate or butyrate supplementation decreases inflammatory gene expression, increases epithelial integrity, and protects the HIE-derived monolayer against invasion by AIEC. (A) qRT-PCR analysis of the indicated enteroid innate immune genes. (B) Change in transepithelial electrical resistance (TEER) of uninfected enteroid monolayers after 4 h of the indicated treatment. TEER at 4 h (T4) was divided by TEER at 0 h (T0). (C) Total numbers of AIEC adherent to and invading the enteroid monolayer. (D) qRT-PCR analysis of the indicated AIEC flagellar and capsule genes. All measurements were performed in DMEM, low glucose alone (CTL) or supplemented with 20 mM propionate (prop) or butyrate (but). The mean of biological triplicates is indicated. Error bars represent the standard deviation. Significance was assessed by a one-way ANOVA with Dunnett’s multiple-comparison test. ****, *P* ≤ 0.0001; ***, *P* ≤ 0.001; **, *P* ≤ 0.01; *, *P* ≤ 0.05; ns, not significant.

Because an anti-inflammatory role for propionate and butyrate has previously been reported *in vivo*, we were surprised to observe a proinflammatory effect for these SCFAs in Caco-2 monolayers (3, 10). We considered that the Caco-2 cell line, which is derived from an adenocarcinoma, might not accurately model the response of the healthy mammalian intestine to SCFAs. To address this, we generated HIE-derived monolayers and measured the impact of SCFAs on expression of a subset of Tlrs and cytokines by qRT-PCR. In contrast to our observations for Caco-2 cells, expression of Tlr5 was significantly decreased by both propionate and butyrate and that of Tlr9, which reduces intestinal inflammation in response to CpG, was increased (Fig. 4A) (20, 21). Expression of the proinflammatory cytokines TNF-α and interleukin 6 (IL-6) was also decreased by propionate and butyrate under select conditions (Fig. 4A). This reduction in inflammatory cytokines was accompanied by an increase in monolayer barrier function as measured by transepithelial electrical resistance (TEER) (Fig. 4B). While propionate and butyrate increased AIEC adhesion and invasion of Caco-2 cells, no effect was observed in HIE-derived monolayers (Fig. 4C). We then questioned whether AIEC shows similar patterns of flagellar and capsular gene transcription in the presence of Caco-2 cells and HIE-derived monolayers. As shown in Figure 4D, propionate and butyrate increased flagellar gene expression and decreased capsular gene expression just as had been observed in Caco-2 coculture. The protective effect of propionate and butyrate on HIE-derived monolayers replicates what has been reported *in vivo* (22–26). This suggests that HIE-derived monolayers are better models of an intact intestinal epithelium than the Caco-2 monolayer.

### Expanding the list of innate immune genes regulated by the SCFAs propionate and butyrate.

**FIG 5:**
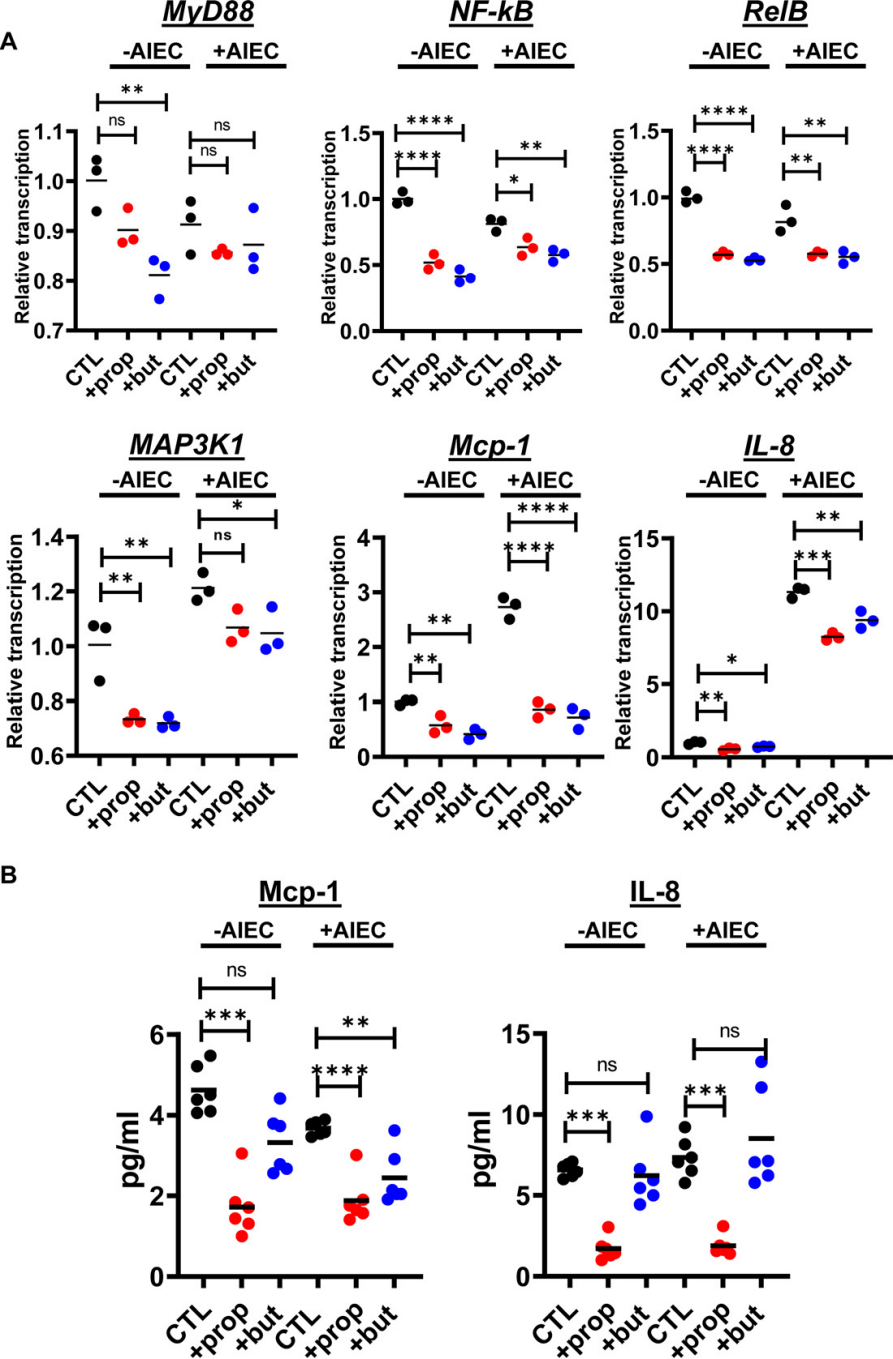
Propionate or butyrate treatment decreases transcription and synthesis of proinflammatory innate immune components in the presence and absence of AIEC infection of HIE-derived monolayers. (A) Relative transcription of the indicated human genes measured using the NanoString nCounter inflammation panel. (B) Concentrations of MCP-1 and IL-8 measured in supernatants in contact with the apical surface of enteroid monolayers as measured using the Legendplex human inflammation panel 1. All measurements were performed in DMEM, low glucose alone (CTL) or supplemented with 20 mM propionate (prop) or butyrate (but). The mean of biological triplicates is indicated for panel A, and the mean of technical duplicates of biological triplicates is indicated for panel B. Error bars represent the standard deviation. Significance was assessed by a one-way ANOVA with Dunnett’s multiple-comparison test. ****, *P* ≤ 0.0001; ***, *P* ≤ 0.001; **, *P* ≤ 0.01; *, *P* ≤ 0.05; ns, not significant.

**FIG 6:**
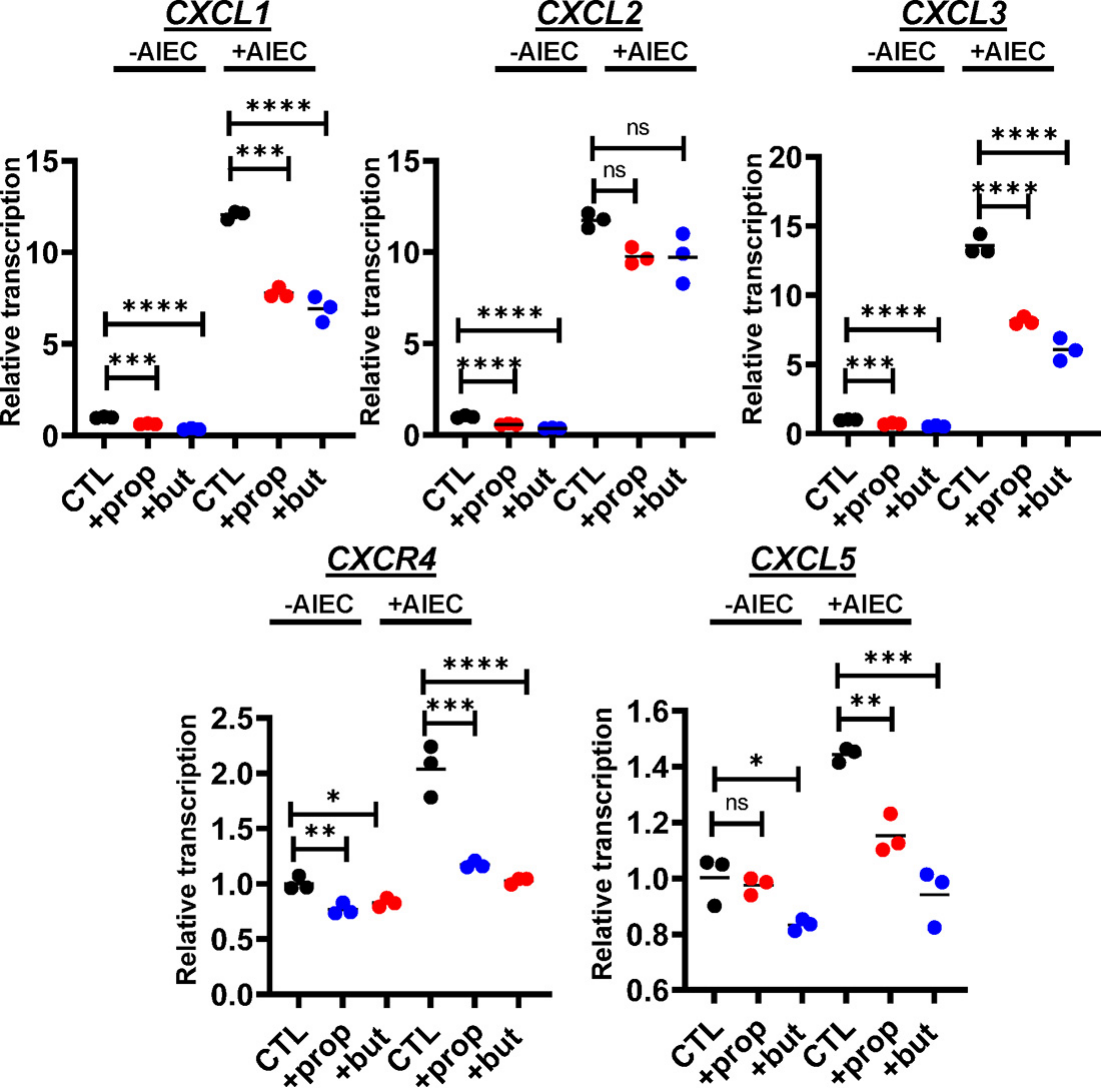
Propionate or butyrate treatment decreases transcription of the proinflammatory cytokine family CXCL in the setting of AIEC infection of HIE-derived monolayers. Relative transcription of the indicated *CXCL* family genes measured using the NanoString nCounter inflammation panel. All measurements were performed in DMEM, low glucose alone (CTL) or supplemented with 20 mM propionate (prop) or butyrate (but). The mean of biological triplicates is indicated. Error bars represent the standard deviation. Significance was assessed by a one-way ANOVA with Dunnett’s multiple-comparison test. ****, *P* ≤ 0.0001; ***, *P* ≤ 0.001; **, *P* ≤ 0.01; *, *P* ≤ 0.05; ns, not significant.

**FIG 7:**
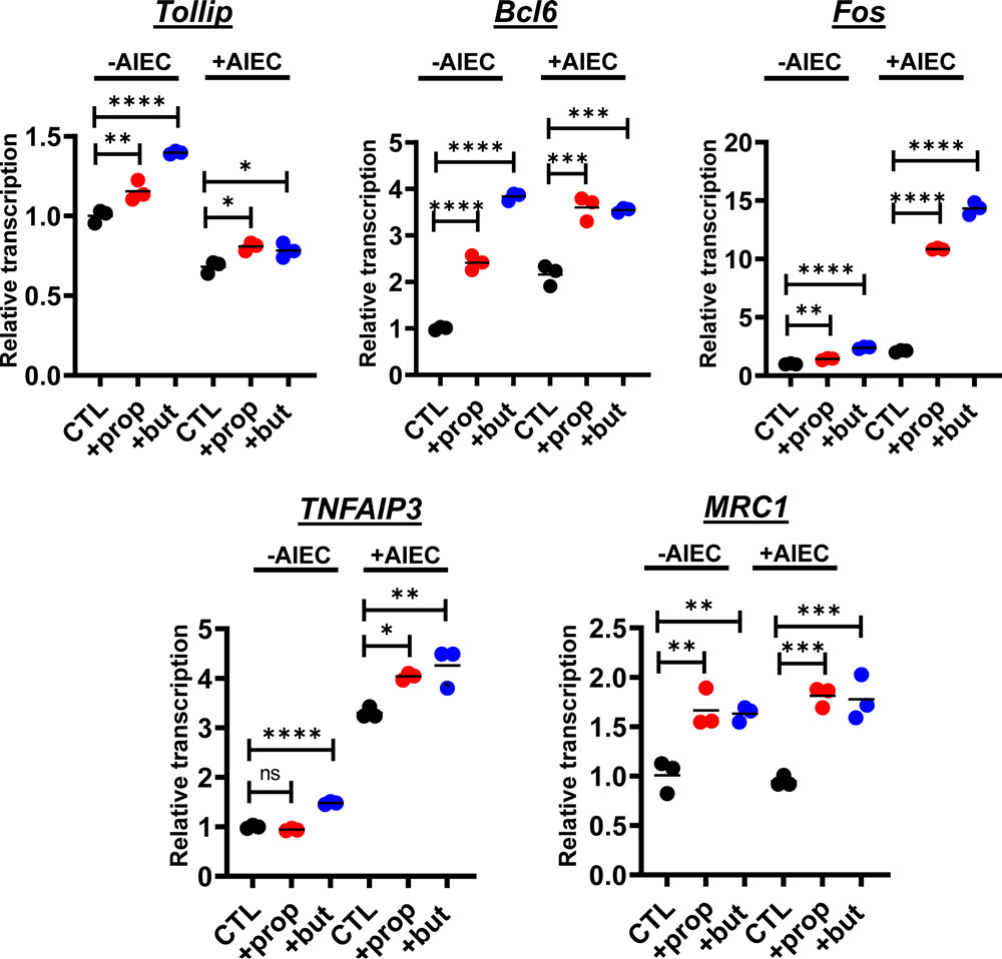
Propionate or butyrate treatment increases transcription of anti-inflammatory genes in both the presence and the absence of AIEC infection of HIE-derived monolayers. Relative transcription of the indicated human genes measured using the NanoString nCounter inflammation panel. All measurements were performed in DMEM, low glucose alone (CTL) or supplemented with 20 mM propionate (prop) or butyrate (but). The mean of biological triplicates is indicated. Error bars represent the standard deviation. Significance was assessed by a one-way ANOVA with Dunnett’s multiple-comparison test. ****, *P* ≤ 0.0001; ***, *P* ≤ 0.001; **, *P* ≤ 0.01; *, *P* ≤ 0.05; ns, not significant.

To further investigate the effects of these SCFAs, we canvassed transcription of a large panel of genes in HIE-derived monolayers in the presence and absence of AIEC. We observed differential transcription of more than 60 genes involved in innate immune signaling, inflammation, epithelial renewal, and carcinogenesis (Fig. 5 and 6 and Table S2). Transcriptional profiles of a representative subset of proinflammatory genes, including *MyD88*, *NF-κB*, *RelB*, *Map3K1*, *Mcp-1*, and *IL-8*, are shown in Figure 5A. Using the Legendplex platform, we also quantified the concentrations of a panel of cytokines in the supernatants of HIE-derived monolayers in response to propionate and butyrate supplementation in both the presence and the absence of AIEC. In agreement with transcriptional measurements, secretion of Mcp-1 was decreased in response to propionate and butyrate (Fig. 5B). However, the trend was less pronounced for butyrate. Secretion of IL-8 was decreased by propionate but not butyrate supplementation. We then formed HIE-derived monolayers in transwells and quantified cytokine secretion from the apical and basolateral aspects of the monolayer. As would be expected of a polarized epithelium, basolateral secretion of both Mcp-1 and IL-8 was much greater than apical secretion (Fig. S2). Both propionate and butyrate decreased secretion of Mcp-1 and IL-8, but again, under most conditions, a significant decrease in IL-8 secretion was observed only in response to propionate supplementation. Transcription of the genes encoding five members of the CXCL family of proinflammatory cytokines was also decreased (Fig. 6). Last, expression of several anti-inflammatory genes was increased with supplementation of either one or both SCFAs (Fig. 7 and Table S2).

## DISCUSSION

Here, we examine the complex relationship between microbially derived SCFAs, the pathobiont AIEC, and the intestinal epithelium. We demonstrate the impact of SCFAs on AIEC adherence to and invasion of immortal and primary intestinal cells, we define the impact of butyrate on the AIEC transcriptome, and we demonstrate the anti-inflammatory effects of propionate and butyrate on primary intestinal cells at the levels of gene transcription and protein expression.

Butyrate, a product of carbohydrate fermentation by anaerobes in particular, is found in all parts of the colon in concentrations of 10 to 30 mM (2, 27). To understand how entry into this portion of the intestine might affect AIEC gene expression, we undertook RNA-seq analysis of AIEC in the presence of 20 mM butyrate. This demonstrated differential regulation of a number of metabolic genes involved in uptake and catabolism of SCFAs. In particular, expression of porins involved in uptake of butyrate decreased significantly, and expression of FadK, which converts butyrate to butyryl coenzyme A (butyryl-CoA), increased. In addition, a coordinate decrease in transcription of genes involved in threonine uptake and propionate catabolism was observed. Threonine is present in DMEM, and its fermentation produces propionate. These changes in transcription, therefore, would be expected to have the overall effect of minimizing the intracellular concentrations of SCFAs. This may allow the bacterial cell to maintain homeostasis in the SCFA-rich environment of the intestine.

Our data suggest that propionate and butyrate supplementation of AIEC also induces a pattern of transcription that maximizes invasiveness. This is supported by previous experiments carried out under different conditions (28). For instance, the AIEC flagellum, whose expression is increased by propionate and butyrate, participates in adhesion to and invasion of the intestinal epithelium (29). Furthermore, several studies have suggested that increased expression of flagellar genes represents AIEC adaptation to the mammalian intestine (17, 30, 31).

Our results also show that the transport and assembly genes of the AIEC type II capsule are downregulated in response to propionate and butyrate. While less is known about the role of the capsule in AIEC pathogenesis, in uropathogenic E. coli (UPEC), a close relative of AIEC, capsule synthesis is downregulated in urine, and this aids adhesion to and invasion of the bladder epithelium (18). Once entry is achieved, the formation of UPEC colonies within bladder cells and survival of dissemination into the bloodstream require capsule synthesis (32, 33). While these two E. coli species become pathogenic in different niches of the mammalian host, for both, environment-specific signals activate invasiveness.

While AIEC virulence genes were upregulated in response to the SCFAs propionate and butyrate in the presence of both Caco-2 and enteroid monolayers, we observed increased intestinal expression of inflammatory cytokines and increased AIEC adhesion and invasion only in the immortalized cancer cell line Caco-2. Inflammation has been reported to facilitate AIEC invasion of cell types such as monocytes and macrophages (31). Furthermore, the deleterious impact of inflammation on the epithelial barrier could provide AIEC access to the basolateral surface of the epithelium (34). We hypothesize that augmentation of the epithelial barrier and suppression of the intestinal inflammatory response by propionate and butyrate protect the intestinal epithelium against colonization and invasion by AIEC.

AIEC colonization and invasion are associated with inflammatory lesions of the intestine caused by inflammatory bowel disease and malignancy. Some have suggested the use of SCFAs as a therapeutic to decrease intestinal inflammation in this setting (3, 35). While we have studied the impact of SCFAs on only one AIEC strain here, there is a large body of literature that suggests that SCFAs serve as a signal to pathogens that they are in the intestine and must increase expression of virulence factors (28, 36–39). Countering this increase in virulence is the anti-inflammatory impact of SCFAs on the intestinal epithelium. In our model, the net impact of SCFAs is to protect the epithelium against AIEC. However, this fine balance is likely influenced by environmental conditions. We conclude that if SCFAs are to be used as anti-inflammatory intestinal therapeutics, their impact on intestinal pathogens must be considered.

## MATERIALS AND METHODS

### Bacterial strains and media.

All infection experiments were performed with AIEC strain LF82, which was a kind gift from Wendy Garrett. AIEC alone was cultured on Luria-Bertani (LB) broth or Dulbecco’s modified Eagle medium (DMEM), low glucose (Gibco 11885084) as noted at 37°C. When the bacteria were cocultured with intestinal cells, the incubation conditions were 5% CO_2,_ at 37°C, for 4 h. Where noted, DMEM, low glucose was supplemented with the indicated concentrations of sodium acetate (BP333500 Fisher Scientific), sodium propionate (A17440 Alfa Aesar), or sodium butyrate (303410 Sigma-Aldrich). LB broth was supplemented with ampicillin 100 μg ml^−1^.

### AIEC growth curves.

AIEC was cultured overnight in LB (Difco 244620) at 37°C, pelleted by centrifugation, and then resuspended in DMEM, low glucose to an optical density at 655 nm (OD_655_) of 1.2. This bacterial suspension was diluted 1:100 in DMEM, low glucose with SCFA supplementation as noted, and 100 μl were dispensed into a 96-well plate in triplicate. The plate was incubated at 37°C in a microplate reader, and OD_655_ was measured every 15 min after agitation (Infinite 200, Tecan).

### Motility and biofilm assays.

Motility assays were performed as previously described with modifications (40). Briefly, AIEC was cultured overnight in LB broth and then stabbed into DMEM soft agar (0.25 % agar) motility plates in triplicate and incubated at 37°C. Motility haloes were measured after 18 h.

For biofilm assays, AIEC was cultured overnight in LB broth. This culture was then used to inoculate 500 μl of DMEM, low glucose in a polystyrene tube in a ratio of 1:100 (vol/vol). Tubes were incubated statically for 24 h at 37°C. Planktonic cells were then removed, nonadherent cells were removed by rinsing with phosphate-buffered saline (PBS), and adherent cells were dispersed by incubation with 500 μl of 1× PBS for 30 min followed by vigorous vortexing for 1 min in the presence of glass beads. The bacterial suspension was added to a 96-well plate, and the OD_600_ was measured.

### RNA-seq.

For transcriptomic analyses, AIEC was grown overnight in LB broth at 37°C, diluted in a ratio of 1:100 in DMEM, low glucose, and incubated for 4 h, at 37°C with shaking. Total RNA was isolated from the bacteria using TRIzol (Invitrogen, 15596018), according to the manufacturer’s instructions. RNA was purified using the Directzol minikit (Zymo R2050) and submitted to Broad Institute Microbial ‘Omics Core for library construction, sequencing, and data analysis (https://www.broadinstitute.org/infectious-disease-and-microbiome/microbial-%E2%80%98omics-core).

### Human cell culture.

The Caco-2 cell line, kindly provided by Dingding An, was cultured at 37°C in a 5% CO_2_ humidified atmosphere in DMEM supplemented with 10% fetal bovine serum and 1% penicillin-streptomycin (Gibco 15140-122). The medium was changed every other day.

Human intestinal enteroids (HIEs) derived from human ileal tissue were purchased from Baylor College of Medicine through the Texas Medical Center Digestive Diseases Center Enteroid Core. HIEs were maintained and passaged as described previously (41, 42). Briefly, HIEs were thawed and resuspended in Matrigel (Corning, 356231). The Matrigel mixture was plated as droplets into 24-well tissue culture plates and incubated at 37°C for 5 to 10 min to allow for polymerization. HIE proliferation medium, consisting of 15% advanced DMEM/F12 (Invitrogen 12634028) supplemented with 100 U/ml penicillin-streptomycin (Invitrogen 15140122), 10 mM HEPES buffer (Invitrogen 15630080), and 1× GlutaMAX (Invitrogen 35050061), 10% Noggin-conditioned medium (collected from Noggin-producing cells that were kindly provided by G. R. van den Brink, Amsterdam, The Netherlands), 20% R-spondin-conditioned medium (collected from R-spondin-producing cells that were kindly provided by Calvin Kuo, Palo Alto, CA), 50% Wnt3A-3 conditioned medium collected from L Wnt-3A cells (ATCC, CRL-2647), 50 ng/ml epidermal growth factor (EGF) (Invitrogen PMG8043), 10 mM nicotinamide (Sigma-Aldrich N0636), 10 nM gastrin I (Sigma-Aldrich G9145), 500 nM A-83-01 (Tocris Bioscience 2939), 10 μM SB202190 (Sigma-Aldrich 7067), 1× B27 supplement (Invitrogen 17504044), 1× N2 supplement (Invitrogen 17502048), and 1 mM N-acetylcysteine (Sigma-Aldrich A9165) were added to each well.

### Monolayer formation from enteroids.

To form monolayers, 3-dimensional enteroids were washed with 500 μl/well of cold 0.5 mM EDTA (Lonza 51201) in PBS (Corning 21040) and collected by spinning at 200 × *g* at 4°C for 5 min. Cells were then dispersed by treatment with 0.05% trypsin at 37°C for 4 min and resuspended in enteroid growth medium containing 10 μM Y-27632 (Sigma-Aldrich Y0503) to obtain cell singlets and doublets. These were seeded on collagen-coated 48-well plates (Corning 354236) or collagen-coated transwells. Monolayers were first cultured in enteroid proliferation medium containing 10 μM Y-27632 for 2 days and then switched to differentiation medium for up to 5 days. Compared with proliferation medium, differentiation medium contains half the R-Spondin and Noggin-conditioned medium and no Wnt 3A, nicotinamide, or SB202190.

### Infections of Caco-2 and enteroid-derived monolayers with AIEC.

AIEC was grown overnight in LB broth, resuspended in DMEM, low glucose, and then added at a multiplicity of infection (MOI) of 10 to human intestinal cell monolayers that had been washed with PBS and cultured with fresh antibiotic-free medium supplemented with 5% inactivated fetal bovine serum for 24 h. Infections were allowed to proceed for 4 h at 37°C, 5% CO_2_ humidified atmosphere.

### Adhesion and invasion assays.

The ability of AIEC to adhere to Caco-2 and enteroid-derived monolayers was assessed using a previously described protocol (43). Briefly, AIEC was cultured overnight in LB broth and then added at an MOI of 10 to monolayers replenished with fresh DMEM. Infected monolayers were then incubated for 4 h, at 37°C in the presence of 5% CO_2_. After the incubation, monolayers were washed, the cells were lysed with 0.1% Triton X-100 in phosphate-buffered saline (PBS; pH 7.4), and the suspension was plated on LB agar to quantify CFU/well. These measurements include both adherent and invasive AIEC. Because the number of adherent cells was always at least 1 order of magnitude greater than that of invasive cells, adherent cells are the main contributor to this measurement.

To measure invasion, monolayers were infected with AIEC at an MOI of 10 for 3 h as described above, treated with 100 μg/ml of gentamicin for 1 h to kill extracellular bacteria, and then lysed with 0.1% Triton X-100. CFU/well was then assessed by plating on LB agar.

### qRT-PCR.

Total RNA was isolated using TRIzol and then purified using the Directzol minikit including an additional DNase I (Zymo E1010) digestion after purification followed by a Monarch RNA clean up kit (T2040). The RNA was reverse transcribed using iScript reverse transcription supermix (Bio-Rad 1708841), and iTaq universal SYBR green Supermix (Bio-Rad 1725121) was used for cDNA real-time PCR amplification, using primer sequences shown in Table S3.

### TEER measurements.

TEER, which quantifies barrier integrity, was measured using a calibrated EVOM2 voltohmmeter. TEER was measured at time zero and 4 h after treatment with the indicated SCFA. The data are presented as the ratio of TEER measured at 4 h to the TEER measured at time zero.

### nCounter NanoString assay.

Expression in monolayer-derived enteroids of 250 inflammation-related genes included in the nCounter human inflammation panel 1 (NanoString Technologies, Seattle, WA, USA) was analyzed using the NanoString nCounter gene expression platform (NanoString Technologies) according to the manufacturer’s protocol. Briefly, 50 ng of total RNA was mixed with a color-coded reporter and a capture probe with target-specific sequences, hybridized overnight at 65°C, and scanned on the nCounter GEN2 digital analyzer. Normalization of raw data and data analysis were performed using the nSolver software 4.0 and nCounter Advanced Analysis 2.0 software (NanoString Technologies).

### Cytokine detection.

Enteroid-derived monolayers were cultured in 48-well plates or transwells as described above. They were treated with SCFAs and infected with AIEC where noted. The supernatants were then collected from the upper compartment for 48-well plates or the upper and lower compartments for transwells and analyzed using the LEGENDplex human inflammation panel 1 (catalog no. 740809, BioLegend, San Diego, CA, USA), according to the manufacturer’s protocol.

### Statistical analysis.

Biological triplicates were performed for all assays and used to calculate the mean measurement, standard deviation, and statistical significance. Statistical analysis was performed using GraphPad Prism Version 4.03 (GraphPad Software). A one-way analysis of variance (ANOVA) or Kruskal Wallis with a *post hoc* Dunnett’s or Dunn’s multiple-comparison test, respectively, was used to calculate statistical significance as appropriate.

### Data availability.

RNA-seq data have been deposited in the NCBI repository (accession no. PRJNA752090).
