# Supplementary material for: The Short-Chain Fatty Acids Propionate and Butyrate Augment Adherent-Invasive Escherichia coli Virulence but Repress Inflammation in a Human Intestinal Enteroid Model of Infection
Source: Microbiol Spectr. 2021 Oct 6;9(2):e01369-21. doi: 10.1128/Spectrum.01369-21 (PMC8510176; doi:10.1128/Spectrum.01369-21)
Supplement: SUPPLEMENTAL FILE 1 — Supplemental material. Download SPECTRUM01369-21_Supp_1_seq11.pdf, PDF file, 0.3 MB [file spectrum01369-21_supp_1_seq11.pdf]

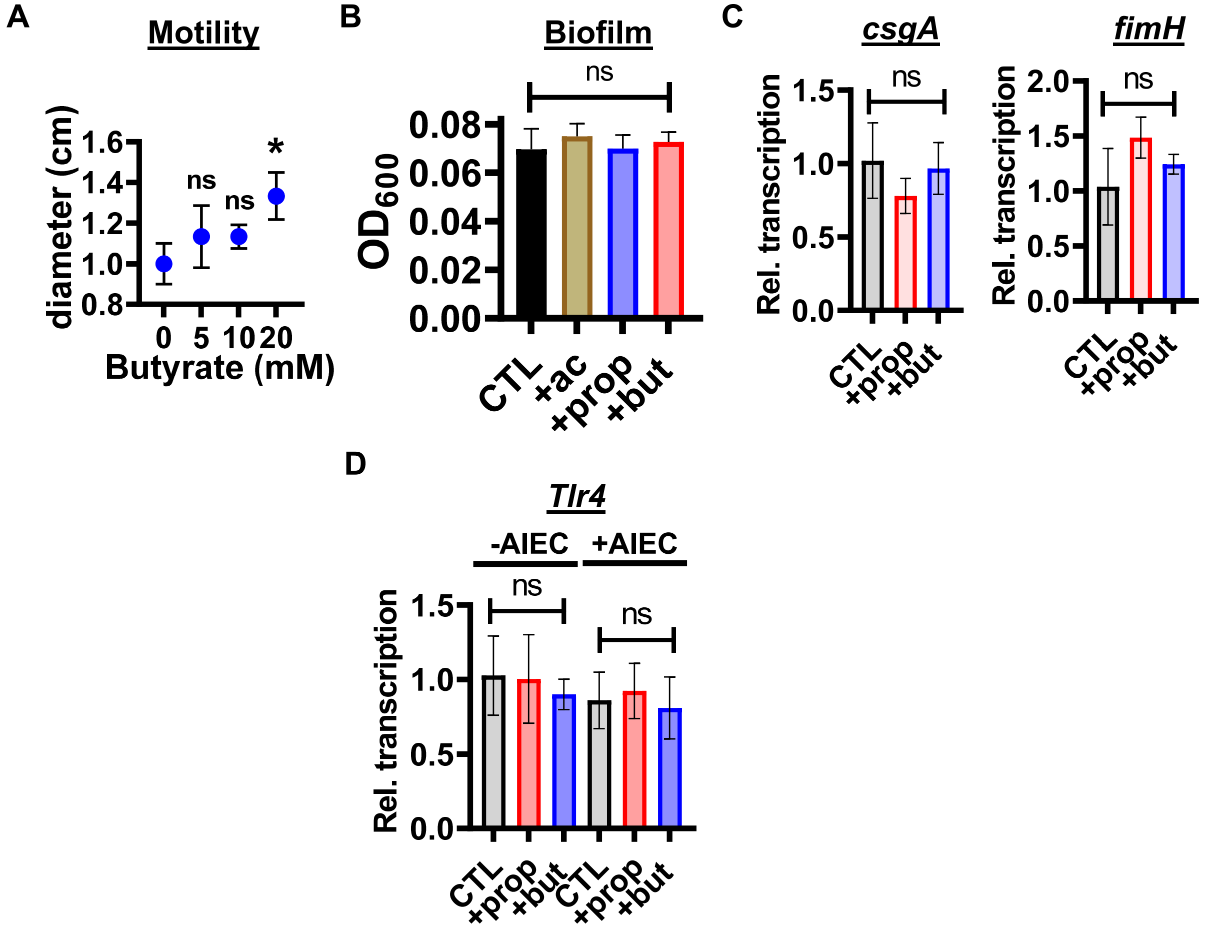

Fig S1: Motility but not Biofilm formation, pilus expression, or Tlr4 expression are differentially regulated by SCFA: (A) Motility in DMEM, low glucose soft agar with butyrate supplementation as noted. (B) OD_{600} representing dispersed biofilms formed in DMEM, low glucose with the indicated SCFA addition. (C) qRT-PCR transcriptional analysis of the indicated AIEC fimbrial genes in co-culture with Caco-2 cells. (D) qRT-PCR analysis of Caco-2 cell *Tlr4* transcription with or without AIEC infection. The mean of biological triplicates is indicated. Error bars represent the standard deviation. Significance was assessed by a one-way ANOVA with Dunnett's multiple comparisons test. * $p \leq 0.05$, ns not significant.

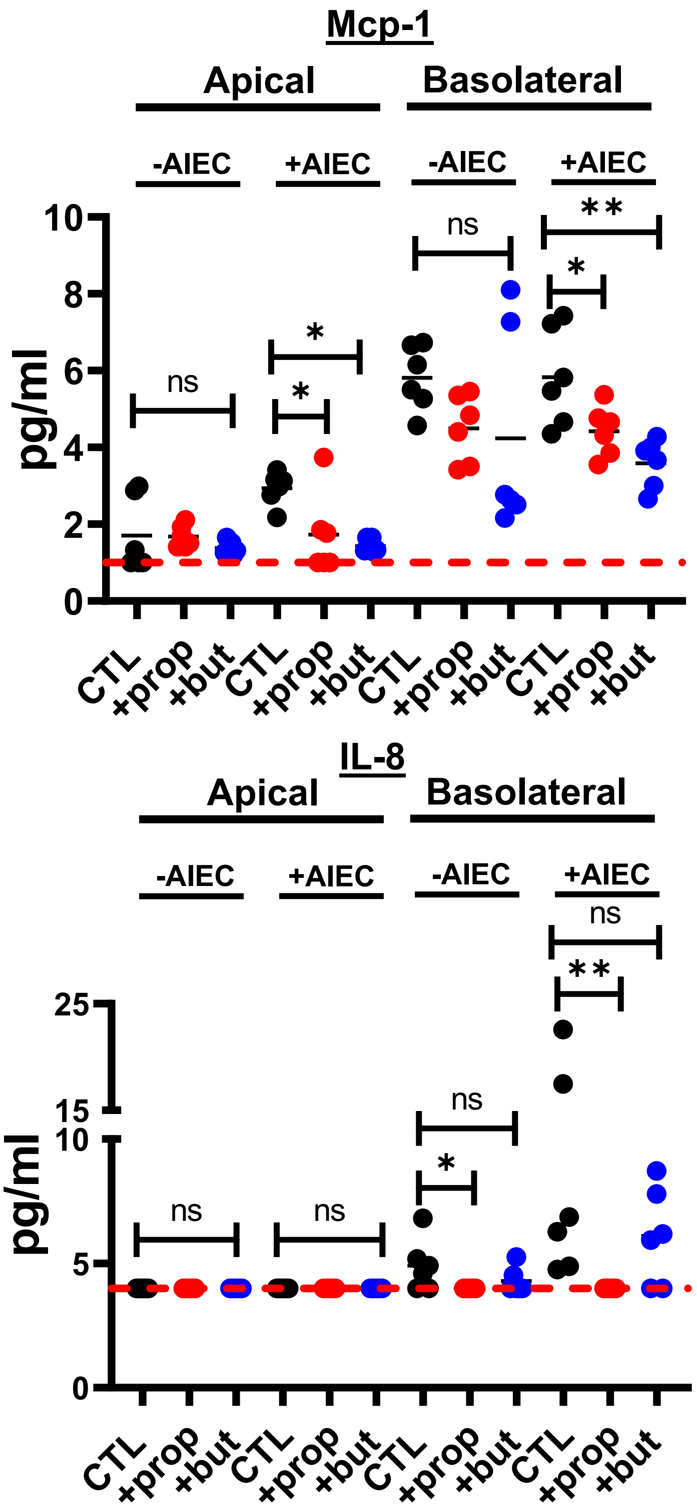

Figure S2: Mcp-1 and IL8 are secreted mainly from the basolateral surface of enteroids, and this is decreased in response to propionate and butyrate. Concentrations of MCP-1 and IL-8 measured in supernatants in contact with the apical and basolateral surfaces of enteroid monolayers cultured in transwells as measured using the Legendplex Human Inflammation Panel 1. All measurements were performed in DMEM, low glucose alone (CTL) or supplemented with 20 mM propionate (prop) or butyrate (but). The mean of technical duplicates of biological triplicates is indicated. Error bars represent the standard deviation. Significance was assessed by a Kruskal-Wallis test with Dunn's multiple comparisons test. ** $p \leq 0.01$, * $p \leq 0.05$, ns not significant.

Table S3: qRT-PCR primers used in this study

TLR-5	F: CCGGGTTTGGCTTCCATAACA<br>R: TGTGAAAGATCCAGGTGTCTCA
TLR-4	F: CGGAGGCCATTATGCTATGT<br>R: TCCCTTCCTCCTTTTCCCTA
TLR-9	F: CTGCCACATGACCATCGAG<br>R: GGACAGGGATATGAGGGATTTGG
TNF-α	F: TCTCGAACCCCGAGTGACAA<br>R: TATCTCTCAGCTCCACGCCA
IL-6	F: CATCCTCGACGGCATCTCAG<br>R: GCTCTGTTGCCTGGTCCTC
rplpo	F: TGGTCATGCAGCAGGTGTTCGA<br>R: ACAGACACTGGCAACATTGCGG
fimH	F: GCGACAGACCAACAACTAT<br>R: TCACGAGCAGAAACATCAC
fliC	F: GTTGCACAGACCACTGAA<br>R: AGAGTTAGTCCCGGTAGAAG
csgA	F: GTAACTCTGCTCTTGCTCTG<br>R: GAGCTGTCAGAACCTTG
rpoA	F: GAGAGTTCAGGGCAAAGATG<br>R: CTGCGGCTTGACGATTT
